# Palliative Care Knowledge and Attitudes of Nursing Home Staff: Baseline Data From the UPLIFT Trial

**DOI:** 10.1093/geroni/igaf122.152

**Published:** 2025-12-31

**Authors:** Kathleen Unroe, John Cagle, Alex Floyd, Wanzhu Tu, Timothy Stump

**Affiliations:** Indiana University School of Medicine, Indianapolis, Indiana, United States; University of Maryland, Batimore, Maryland, United States; Regenstrief Institute, Indianapolis, Indiana, United States; Indiana University Indianapolis, Indianapolis, Indiana, United States; Indiana University Bloomington, Bloomington, Indiana, United States

## Abstract

Palliative care and hospice are known to improve quality of care within nursing homes. However, such care is underutilized in long term care settings. One known barrier is staff knowledge and attitudes. To evaluate the palliative care knowledge, attitudes, and practices among nursing home staff, we surveyed (n = 242) frontline nursing home staff in 16 facilities located in two states, n = 8 in Maryland and n = 8 in Indiana, using a validated survey instrument. The survey contains domains including family communication, provider communication, planning/intervention, and bereavement, and utilizes patient vignettes. Participating nursing home communities were eligible if they: had >40 long stay residents with cognitive impairment and did not have an active in-house palliative care program. Surveys were collected in person by research staff. Survey completion varied across facilities (from n = 6 to n = 23). Participating nursing home staff had an average age of 43 years old, 92% female, and 49% were Black/African American. The most common job categories for nursing home staff completing the survey were certified nursing assistants (44%), followed by nurses including LPNs (20%, and RNs (8%). With higher scores indicating greater knowledge/better attitudes (possible range 1-4 on each item), we found that participating staff had: M = 3.1 (SD = 0.6) on the Family Communication subscale; M = 2.9 (SD = 0.8) on the Provider Coordination subscale; M = 2.7 (SD = 0.9) on the Planning/Intervention subscale; and M = 2.4 (SD = 0.6) on the Bereavement subscale. Overall, palliative care knowledge scores for physical symptoms were higher than psychological symptoms, which were both higher than knowledge of end of life.

